# Modulation of CREB and its associated upstream signaling pathways in pesticide-induced neurotoxicity

**DOI:** 10.1007/s11010-022-04472-7

**Published:** 2022-05-21

**Authors:** Rekha Koravadi Narasimhamurthy, Daicy Andrade, Kamalesh Dattaram Mumbrekar

**Affiliations:** 1 Department of Radiation Biology and Toxicology, Manipal School of Life Sciences, Manipal Academy of Higher Education, Manipal, 576104 Karnataka India; 2 Manipal School of Life Sciences, Manipal Academy of Higher Education, Manipal, Karnataka India

**Keywords:** Pesticides, Neurotoxicity, CREB, BDNF, AKT, PI3K

## Abstract

Human beings are exposed to various environmental xenobiotics throughout their life consisting of a broad range of physical and chemical agents that impart bodily harm. Among these, pesticide exposure that destroys insects mainly by damaging their central nervous system also exerts neurotoxic effects on humans and is implicated in the etiology of several degenerative disorders. The connectivity between CREB (cAMP Response Element Binding Protein) signaling activation and neuronal activity is of broad interest and has been thoroughly studied in various diseased states. Several genes, as well as protein kinases, are involved in the phosphorylation of CREB, including BDNF (Brain-derived neurotrophic factor), Pi3K (phosphoinositide 3-kinase), AKT (Protein kinase B), RAS (Rat Sarcoma), MEK (Mitogen-activated protein kinase), PLC (Phospholipase C), and PKC (Protein kinase C) that play an essential role in neuronal plasticity, long-term potentiation, neuronal survival, learning, and memory formation, cognitive function, synaptic transmission, and suppressing apoptosis. These elements, either singularly or in a cascade, can result in the modulation of CREB, making it a vulnerable target for various neurotoxic agents, including pesticides. This review provides insight into how these various intracellular signaling pathways converge to bring about CREB activation and how the activated or deactivated CREB levels can affect the gene expression of the upstream molecules. We also discuss the various target genes within the cascade vulnerable to different types of pesticides. Thus, this review will facilitate future investigations associated with pesticide neurotoxicity and identify valuable therapeutic targets.

## Introduction

Pesticides are extensively used globally to destroy weeds (herbicides), rodents (rodenticides), insects (insecticides), fungus (fungicides), or other harmful organisms, thereby aiding human beings in the industrial, agriculture, and health-care sectors. Due to their pervasiveness, an individual can be exposed to pesticides through the intake of contaminated water, pesticide-poisoned air, and dust debris on vegetables and fruits, fatty tissue of animals exposed to the pesticide along with their by-products (i.e., eggs, meat, and fish), occupational exposure during pesticide production and living in areas with immense pesticide residue [1]. Since pesticides are not always selective, exposed individuals develop acute and chronic effects in different organs [2]. Pesticide exposure is associated with various conditions like cancer, neuropathy, axonopathy, asthma, hypersensitivity, metabolic, and developmental disorders [1]. In addition, different pesticides, such as insecticides, including organophosphate, organochlorines, and carbamates, have the potency to cause neuronal damage [3]. The internal features of the nervous system, like axonal transport, neurotransmission process, myelination of neurons, and formation of synaptic processes, have higher vulnerability to a toxic insult when exposed [4].

Exposure to these neurotoxic agents also provokes changes in different gene expression and signaling pathways, manifesting various neurotoxic effects. The cAMP Response Element Binding Protein (CREB) family of transcription factors is one of the critical regulators of neuronal differentiation, survival, and plasticity through their involvement in the BDNF-TrkB (Tropomyosin receptor kinase B) pathway. It is well known that BDNF, a vital neurotrophin, aids in the survival of extant neurons, strengthening the development of new neurons, promoting neuronal plasticity, migration, differentiation, neurite growth, synapse formation, and potentiation [5, 6]. Further, CREB activation also results through various kinases like PKA (Protein kinase A), Ras, ERK (Extracellular-regulated kinase), and MAPK (Mitogen-activated protein kinase) family members. This review attempts to elucidate the mechanism of activation of CREB through various CREB kinases and their fundamental role in pesticide-induced neurotoxicity integrally.

## Pesticides and neurotoxicity

Neurotoxicity is the neurophysiological alteration due to exposure to toxicants leading to cognitive and memory impairment and may lead to psychiatric disorders [7]. Pesticides can lead to inadvertent neurotoxicity in humans due to the similarity in the acetylcholinesterase enzyme structure with insects [4]. Inhibition of acetylcholinesterase leads to over aggregation of acetylcholine at the neuronal junction, resulting in synaptic transmission blockage and subsequent neurotoxicity [8]. The two major classes of pesticides that interfere with acetylcholine release are organophosphates (malathion, chlorpyrifos, parathion, diazinon, and dichlorvos) and carbamates (methylcarbamate, polyurethane, and ethyl carbamate) [9, 10]. Compared to organophosphates, carbamate inhibition of the enzyme acetylcholinesterase is not permanent and can be easily adjustable [11]. However, acute exposure to high- or low-dose chronic exposure can result in severe or delicate neurotoxicity symptoms by inhibiting acetylcholine esterase enzyme and other non-cholinergic symptoms [2, 12]. Apart from the acetylcholinesterase inhibition, neurotoxicity can occur through several other malfunctions, including neuropathy, axonopathy, myelopathy, and ultimately affecting neurotransmission [4].

**Fig. 1:**
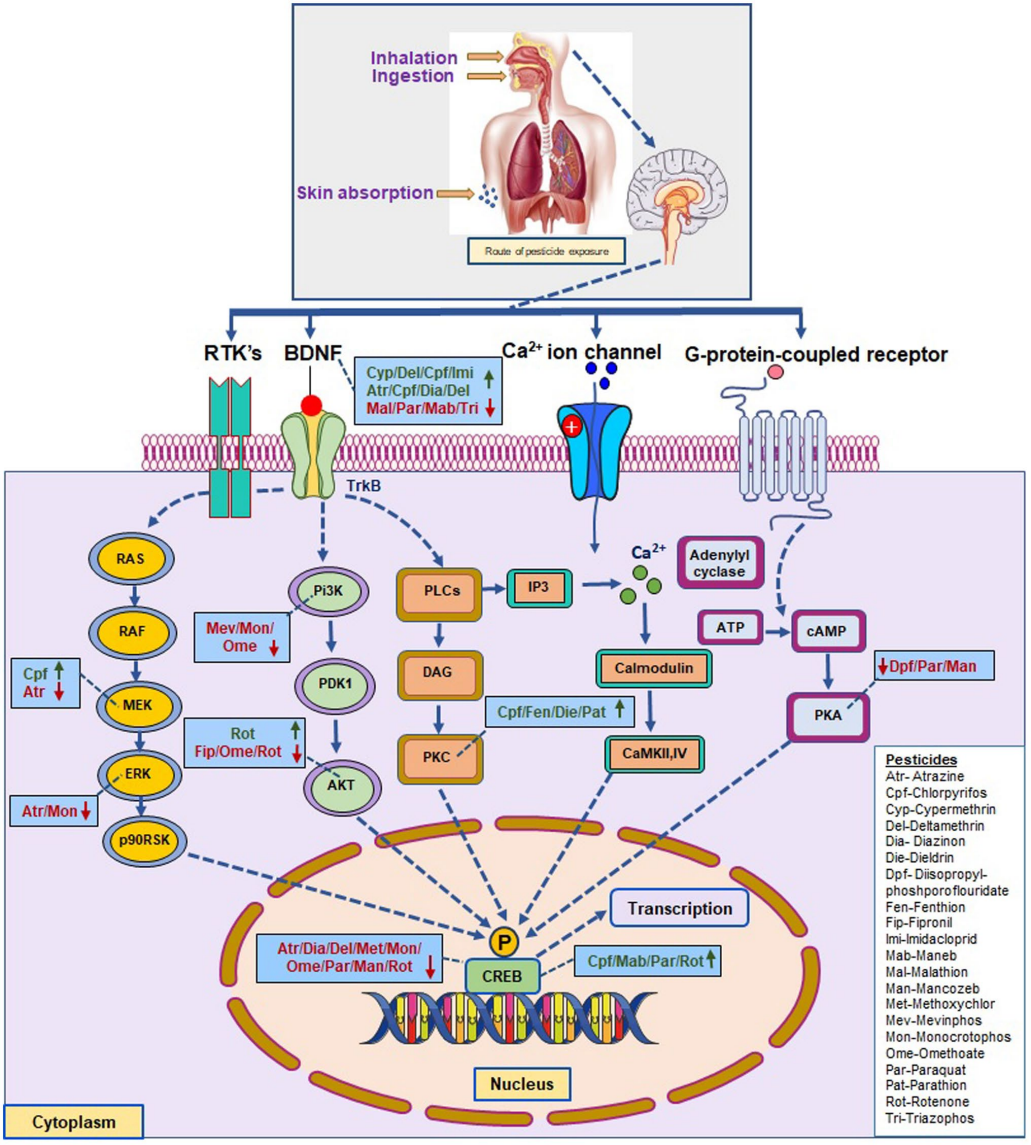
Pesticides on entering the body through inhalation, ingestion, and skin absorption can reach the brain due to their lipophilic nature. Upon entering the brain, it can target the components within the various signaling pathways like BDNF/TrkB pathway, RAS/RAF/MEK pathway, Pi3K/AKT pathway, PLC/PKC pathway, or through the Calcium–Calmodulin pathway and cAMP pathway and ultimately affect CREB phosphorylation and gene expression. The changes in the phosphorylation or gene expression can, in turn, affect the various functions of CREB, including the regulation of neuronal plasticity and survival. [The scientific diagram was constructed using Servier Medical Art (SMART), licensed under a Creative Commons Attributio 3.0- https://smart.servier.com]

**Fig. 2:**
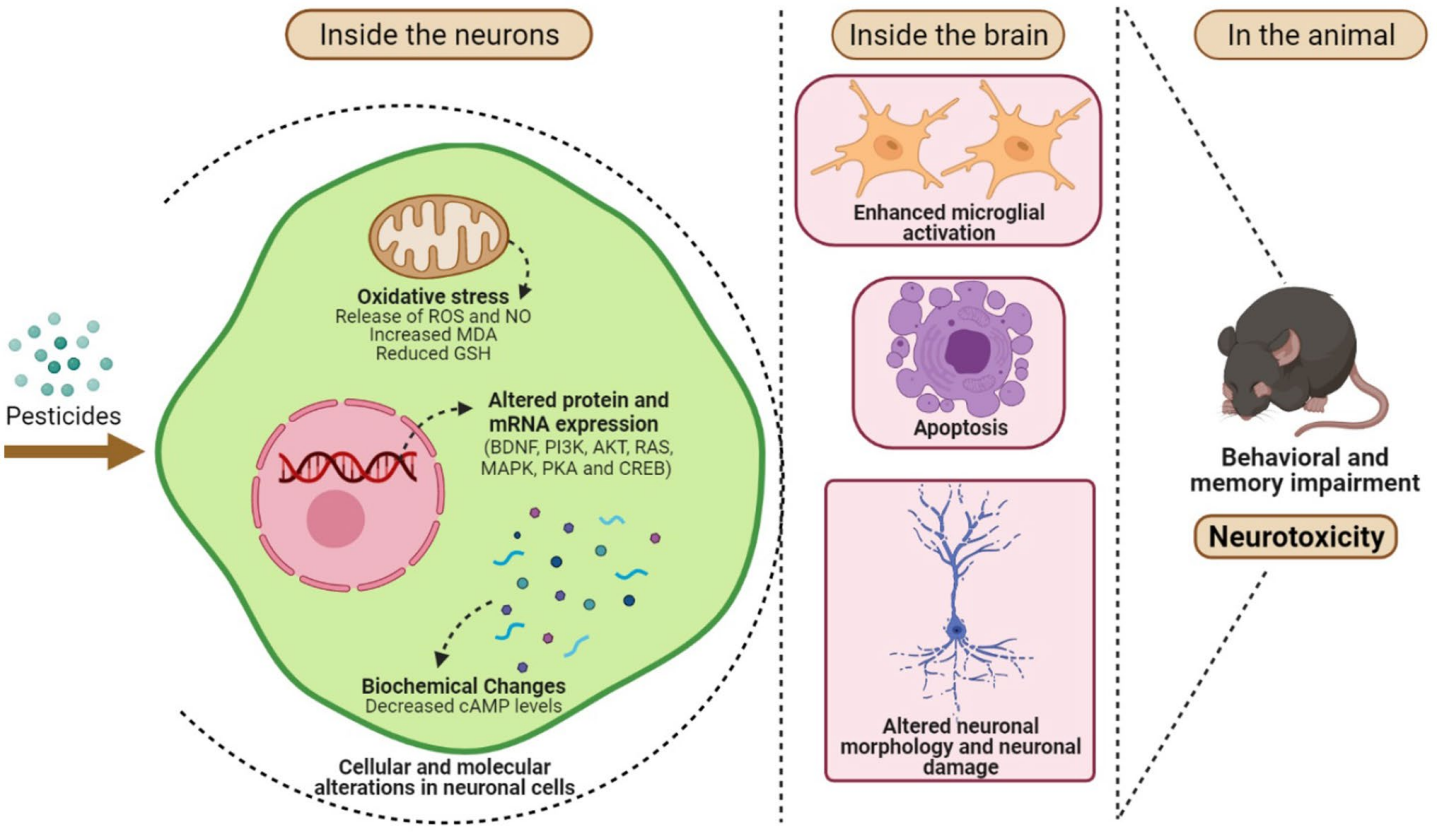
Gross summary of cellular and molecular changes accompanying the modulation of CREB-related pathways and their outcomes in the pesticide-exposed individuals. Pesticide exposure can lead to cellular and molecular alterations within the neurons while also enhancing microglial activation, damaging the neuronal morphology, and sometimes leading to apoptosis. These changes most often present themselves in the form of immobility, cognitive damage, and impaired learning and memory in the exposed individual. (Created with BioRender.com.)

Organophosphate pesticides can cause neuropathy, apoptosis, or necrosis of neurons, resulting in progressions of neurodegenerative disorders like Parkinson’s and Alzheimer’s [2, 4]. Axonopathy results when pesticides like chlorpyrifos and rotenone interfere with axon activity, leading to weak motor strength, difficulty in sensation, resulting in axonopathy [13]. Pesticides like chlorpyrifos and cypermethrin can also lead to myelopathy by disturbing axon myelination [14]. The various neurotoxic effects of the different pesticides are listed (Table 1), and the pathways affected are depicted in Fig. 1. Further, the overall neurotoxic effects are also grossly summarized in Fig. 2.

**Table 1:** Effect of various insecticides on CREB and related upstream signaling molecules:

Pesticide	Organism	Dose	Effects	Reference
Atrazine	Male Sprague–Dawley (SD) rats (2 months old)	25 or 50 mg	Increased BDNF expression	[38]
Atrazine	SD rats	10 or 100 mg/kg body weight every day for 30 days from PND 35	Impaired memory and damaged DG and CA1 neurons, downregulated protein and mRNA expression levels of MEK/ERK/CREB and BDNF	[50]
Chlorpyrifos	Primary cortical and hippocampal neurons from rat pups (E18)	Doses ranging from 0 to 10 µM for 1 h	CREB phosphorylation increased	[28]
Chlorpyrifos	Male Wistar rats	10 mg/kg body weight	Reduced BDNF levels	[47]
Chlorpyrifos	PC12 cells	25, 50, 100, and 200 μM for 24 h	MEK activation leads to the generation of ROS and neuronal apoptosis	[15]
Chlorpyrifos and fenthion	Female SD rats and PC-12 cells	82 mg chlorpyrifos/kg body wt., 108 mg fenthion/kg body wt., 40 mg DDT/kg body wt. 0, 50, 100, or 200 nM pesticides in PC 12 cells	PKC activation in the brain region leads to oxidative damage	[89]
Chlorpyrifos, Diazinon	Neonatal SD pups (PND 1–4)	1 mg/kg and 1 or 2 mg/kg respectively	Decrease in the *creb1* expression with 2 mg/kg Diazonin	[30]
Chlorpyrifos, Diazonin/PC12 cell line	SD rats aged PND 1–4	1 or 2 mg/kg body weight 30 µM	Reduced BDNF in undifferentiated cells	[46]
Cypermethrin, Deltamethrin, Chlorpyrifos, and Imidacloprid	Zebrafish brain	0.0024 µM 0.29 µM, 2 µM and 45 µM respectively	Elevated levels of BDNF transcription	[40]
Deltamethrin	Primary cortical neuron culture out of 17-day old SD rat embryos	1 µM	Increased BDNF expression and thus neurite outgrowth	[39]
Deltamethrin	Female SD rats	0, 0.54, 1.35, and 2.7, 9 mg/kg body weight	Impaired cognitive ability and decrease in BDNF, pTrkB/TrkB, and p-CREB/CREB expression levels in the hippocampus	[41]
Dieldrin	Rat mesencephalic dopaminergic cells	60 μM for 30 min	Upstream signaling of PKC mediated apoptotic cell death	[91]
Diisopropyl-phosphorofluoridate	White leghorn layer hens	1.7 mg/kg body weight	Decreased levels of PKA and p-CREB in cerebrum nuclear fraction	[34]
Fipronil	Human neuroblastoma cell lines	25,50 and 100 μM for 24 h	Inactivation of AKT expression in a concentration-dependent manner	[57]
Malathion	Adult male albino rats	1132.5 mg/kg body weight	Reduced BDNF levels	[45]
Mancozeb	*Thalassoma pavo* female adult fishes	0.2 mg/L	Activation of p-CREB, decreased exploration, latency to reach T-maze arms, and immobility	[29]
Methoxychlor	Female CD-1 mice	16, 32 or 64 mg/kg body weight	Increased levels of p-CREB in mitochondria	[35]
Mevinphos	Rostral ventrolateral medulla of adult male SD rats	10 nmol	Pi3K activation is prevented	[63]
Monocrotophos	Wistar rats/Neural stem cells isolated from embryonic day 12 rat fetuses	10 mg/kg body weight single dose/10 micromolar	Downregulated expression of TrkA, pERK ½, pAkt, and p-CREB both in vitro and in vivo with behavioral impairments seen in rats	[32]
Monocrotophos	Human mesenchymal stem cell	10, 100, 1000 μM for 24 h	Pi3K gene expression is decreased	[66]
Omethoate	Adult male Kunming mice	5 mg/kg was injected for 4 consecutive weeks	Expression of p-CREB, p-PI3K, and p-Akt decreased caused increased immobility and neuronal damage	[19]
Paraquat	Mesencephalic rat cells	100, 250, 400 µM	Increased expression of p300/CREB	[43]
Paraquat	IFN-γ knockout (KO) mouse	10 mg/kg body weight 3 times a week for 3 weeks	Transient suppression of CREB and BDNF	[44]
Paraquat and Maneb	SD female pregnant rats	10 or 15 mg/kg body weight twice a week from 6th gestational day till ablactation	Behavioral impairment, altered hippocampal neuron morphology, Reduced PKA production, and CREB phosphorylation	[48]
Paraquat and Maneb	SD male rats	5, 10, 20, 35 mg/kg bodyweight for 12 weeks	Reduced PKA production, reduced levels of CREB, BDNF, and p-CREB	[49]
Parathion	Primary cerebellar neuronal cultures from SD rat pups	200 μM for 0–120 min	Activation of PKC leading to caspase-3 neurotoxicity	[92]
Rotenone	Male Wistar rats	1.2 mg/kg body weight subcutaneous injection	Decreased levels of p-CREB with the release of ROS and NO	[27]
Rotenone	Wistar rat pups	0.1 mg and 0.5 mg per kg body weight	Decreased CREB expression on PND 12 (0.1 and 0.5 mg), PND 60 (0.5 mg) and decreased CBP expression on PND 30 (0.5 mg), PND 60 (0.1 mg, 0.5 mg)	[33]
			Increased CREB expression on PND 30 (0.1 mg), PND 120 (0.5 mg), and increased CBP expression on PND 120 (0.1 mg and 0.5 mg)	
Rotenone	Rat adrenal pheochromocytoma cells	0.1, 0.5, 1.0, 5.0 and 10 μM for 24 h	Inhibition of AKT expression	[58]
Rotenone	Adult mice	1 mg/kg or 3 mg/kg for 21 consecutive days	Activation of AKT reduced the Neurotoxicity of Rotenone	[60]
Rotenone	Human neuroblastoma cell line	10 µM rotenone for 24 h	CREB phosphorylation decreased	[22]
Rotenone	SH-SY5Y cells	200 nM	Induction of phosphorylation of JNK, p38MAP kinase	[73]
Rotenone	Male Wistar rats	1.5 mg/kg for 3 weeks	Upregulation of p38MAPK with enhanced microglial activation and induction of neuronal apoptosis	[82]
Triazophos	Wistar male albino rats	8.2 mg/kg body weight orally daily for 30 days	Cognitive impairment, Reduced GSH, Increased MDA, and Reduced expression of BDNF	[42]

### Regulation of CREB signaling

CREB is a crucial member of the leucine-zipper family of structurally and functionally similar transcriptional regulators and is essential for neuronal functioning, development and maintenance, and long-term synaptic plasticity [16]. The activation of CREB is carried out by the phosphorylation of its Ser133 residue in the presence of co-activator molecule CREB-binding protein (CBP) through various kinases like PKA, mitogen-activated protein kinase 2 (MAPK2), ribosomal S6 kinase 2 (RSK2), Ca^2+^-activated calmodulin kinases (CAMK), etc. [17]. CREB can also be activated to initiate specific upstream signaling pathways like the BDNF-Trkb pathway through the mediation of protein kinases [18]. Thus, the activation of CREB results from the convergence of multiple signaling cascades involving several different protein kinases, each having its role in regulating neuronal activity and functions.

The CREB is critical in developing the nervous system and controls multiple target genes involved in neuron development, circadian rhythms, depression, survival, excitability, regulating neuron plasticity, formation of synapsis, axon growth, and long-term potentiation [19, 20]. CREB activation underlines diverse adaptive development critical for neurotrophin-mediated survival of neurons against oxidative damage or inflammation mediated toxicity [21, 22]. The activated CREB is then recruited to carry out the transcription of other genes like Bdnf, Akt, etc., in the neuronal cells, essential for several complex and dynamic neuronal functions, including plasticity, synaptic transmission, neuronal development, survival, and their neurotrophic regulation [16].

The alterations in the CREB phosphorylation levels are linked to decreased cAMP levels leading to protein kinase A-mediated CREB phosphorylation [23]. Several pesticides affect the CREB levels by targeting it directly or its upstream signaling cascade [24]. Decreased expression of CREB due to direct interaction of organophosphate pesticides may lead to the chronic low-level onset of pesticide neurotoxicity and affect the transcription of genes correlated with learning and synaptic plasticity [25, 26]. Further, the decreased levels of p-CREB (phosphoCREB) also accompanies the release of ROS and NO in rotenone-administered rats [27]. Studies have shown that elevated phosphorylated CREB levels exhibit favorable outcomes in the exposed individual. Following that observation, an increase in the p-CREB level was found in the cortical and hippocampal neurons after low-dose chlorpyrifos exposure, possibly displaying neuroprotective effects [28]. Mancozeb, a potent pesticide, also showed a notable activation of p-CREB, attributing to early neuroprotection [29]. A dose-dependent fall in the *Creb1* expression was also noticed in the brain regions of animals treated with diazinon [30]. In comparison, another study showed that reduced expression of (CaMK)-IV and CREB1 mRNA levels contributed to the impaired novel object recognition in mice [31].

Further, monocrotophos treatment resulted in the decreased level of p-CREB along with the associated upstream molecules, namely pERK1/2, p-AKT, and pTrkA (Tropomyosin receptor kinase A), leading to apoptosis and neuronal injury [32]. Rotenone exposure in rats showed alterations in CBP (CREB-binding protein) and CREB levels, with a significant decrease seen in several treatment groups, manifested as behavioral and synaptic protein abnormalities [33]. The differential alterations in the PKA/p-CREB pathways culminated in gross cytoskeletal damage in the central nervous system in hens treated with diisopropyl phosphor fluoridate (DFP) [34]. Interestingly, the immunoreactivity of phosphorylated mitochondrial CREB was found to increase upon methoxychlor exposure in response to oxidative stress [35].

Although pesticides can directly target CREB expression levels, the upstream activation of CREB includes several key members of different signaling pathways like BDNF/Trk, Pi3K/AKT, RAS/MEK/ERK, PLC/PKC, etc., which also make them vulnerable to pesticide insult. Alterations in the expression levels of these genes/proteins can affect the neuronal functions associated with the CREB interference and are further discussed below.

### CREB and BDNF

BDNF, one of the most important neurotrophic factors essential for neuronal functioning and survival, modulates its function by mediating the CREB transcription factor. The interaction of BDNF by selectively binding to tyrosine kinase B (TrkB) at residues Tyr490 and Tyr515 results in homodimerization and provokes the activation of adaptor proteins such as Src homology domain 2 (SH2) and polypyrimidine tract-binding protein (PTB). These stimulated adaptor proteins generally activate three cascading intracellular RAS/MEK, Pi3K/AKT, and PLC/PKC signaling pathways [36]. Both RAS and Pi3K signaling regulates the neurotrophic activity of survival and growth through activating the transcription factor CREB, resulting in protein-dependent synaptic plasticity through activation of BDNF expression [37].

Many studies have shown that changes in the BDNF-TrkB pathway can disrupt the physiological process, cause cognitive deterioration and neurotoxicity [5]. A decrease in the CREB phosphorylation influences BDNF/TrkB signaling pathway and enhances oxidative stress and neurodegeneration [20]. The BDNF expression is altered in the early stages of Parkinson's disease in atrazine-induced neurotoxicity [38]. Further, it is also proved that mutations that disrupt the binding of CREB can, in turn, reduce BDNF responses demonstrating the interplay between these two signaling molecules [21]. Deltamethrin, an insecticide that belongs to the pyrethroid family, increases neurite outgrowth in cortical neurons by activating the endogenous BDNF/TrkB pathway[39]. Cypermethrin, chlorpyrifos, deltamethrin, and imidacloprid exposure upregulates the mRNA and protein levels of BDNF in the brain of adult zebrafish [40]. On the contrary, pyrethroid and deltamethrin exposure in rats decreased the expression of BDNF, pTrkB/TrkB, and p-CREB/CREB levels [41]. Triaophos-administered rats exhibited lower levels of BDNF in the hippocampi and presented deficits in learning and memory accompanied by oxidative stress [42]. Paraquat exposure can induce cell death through the increased expression of p300/CREB-binding protein (p300/CBP) and phosphorylates p53 [43]. Transient suppression of BDNF and CREB was also observed upon paraquat administration [44].

Malathion, one of the most commonly used organophosphates, showed a significantly reduced BDNF level and apoptosis in female rats [45]. Studies have also shown a significant reduction in the transcript of *Bdnf-Trkb* in rats exposed to organophosphate pesticides such as diazinon and chlorpyrifos [46, 47]. Combined exposure to paraquat and maneb reduced PKA production through cAMP stimulation and, thus, inhibited activating elements like BDNF, CREB, ARC, C-JUN, C-FOS, etc.[48, 49]. Further, BDNF downregulation eventually reduces the *Creb* mRNA and protein levels through the MER/ERK pathway [50]. Changes in the BDNF-TrkB pathway, thus, disrupts the physiological process, induces cognitive deterioration, and neurotoxicity. However, a study has shown that estrogen, in a CREB- independent mechanism, also activated B*dnf* expression by interacting with the BDNF promoter, though this is not predominantly observed [51]. Thus, the transcriptional regulation of BDNF is dependent on the successful phosphorylation of CREB.

### CREB and the Pi3k/AKT

CREB activation is well known to promote cell survival by modulating anti-apoptotic genes, enhancing neurotrophin levels, and combating oxidative stress by stimulating various antioxidant genes [52]. However, at times, changes in the expression levels of other essential genes can affect CREB and modulate neuronal survival. AKT, a serine/threonine-specific kinase, and its isoforms are expressed mainly in the brain and have an essential role in neuroprotection, preventing neurodegenerative disorders and oxidative damage. AKT activation is affected by many factors, such as growth factors, cellular stress, and cytokines. The Pi3K/AKT pathway blockage leads to the loss of phosphorylated AKT levels, thus, leading to neurotoxicity [53, 54]. AKT signaling prevents oxidative stress by activating nuclear factor erythroid-derived 2-like 2(NRF2), eventually preventing neurotoxicity [55].

It was shown that fenitrothion and fenitrothion-oxon reduced the phosphorylation of CREB and AKT while chlorpyrifos reduced the phosphorylation of ERK2, p90RSK along with CREB and AKT [56]. CREB and AKT phosphorylation were downregulated in the hippocampus after exposure to omethoate insecticide, accompanied by increased immobility in behavioral tests and neuronal damage. CREB downregulation could be partly reversed by targeting a therapeutic strategy against it, indicating that CREB manifests a protective effect on the neurons and is essential for their survival[19]. Rotenone-induced neuronal apoptosis was observed in the human neuroblastoma cell line, showing reduced phosphorylated CREB and AKT levels [22]. Further, studies have shown that inhibition of AKT in a concentration-dependent manner due to insecticide exposure results in neuronal cell damage [57]. Accordingly, fipronil, a phenylpyrazole insecticide, promotes apoptosis in neuroblastoma cells by blocking the phosphorylation of the AKT [57]. Further, rotenone insecticide induces neurotoxicity by causing apoptosis in dopaminergic neurons by preventing *Akt* gene expression [58]. These exposures may result in the onset of neurodegenerative symptoms as it is well known that defective *Akt* expression is linked to reduced dopaminergic neurons in Parkinson’s disease [59]. It has been reported that the activation of the AKT cascade resulted in reduced neurotoxicity of rotenone [60]. Since AKT primarily interacts with the CREB transcription factor, it can hamper the gene expression in the exposed individual [56]. AKT activation promotes anti-apoptotic signals against neuronal cell death induced by neurotoxins and can contribute toward neuroprotective effects that provide the basis for new therapeutic targets for alleviating neurotoxicity.

Pi3k/AKT pathway is essential for negotiating neuronal survival and crucial in long-term neuronal potentiation occurring upstream of CREB. Pi3K/AKT pathway is involved in numerous diseases associated with oxidative stress and is dysregulated under neurotoxic conditions [61]. Evidence has also proven that rotenone induces dopaminergic degeneration by altering the Pi3K/AKT pathway [22]. In addition, activation of Pi3K/AKT signaling in rostral ventrolateral medulla during mevinphos organophosphate intoxication results in impairment of brain stem cardiovascular regulation that underpins circulatory depression [62, 63].

Phosphoinositide 3-kinases (Pi3K), one of the CREB activating kinases, are widely expressed in the mammalian brain and are involved in growth, proliferation, differentiation, and play an essential role in neuronal survival by regulating metabolism, preventing apoptosis, and learning and memory formation [64]. The activation of Pi3K has been correlated with the transference of anti-apoptotic signals and cytoprotective effects against neurotoxicity [65].

A recent study has shown that Pi3K mediates neuronal survival activity in monocrotophos organophosphate-induced neurodegeneration in human tissues [66]. The hindrance of the Pi3K pathway leads to an increase in apoptosis reaction in the central nervous system of neurodegenerative patients due to activation of pro-apoptotic proteins such as BAD. A correlation is seen between hippocampal neuron apoptosis with the reduction of anti-apoptotic protein expression due to the hindrance of the Pi3K cascade in the Pi3K pathway resulting in neuronal apoptosis. At the same time, further upregulation of Pi3K was shown to inhibit rotenone-induced neurotoxicity [22]. Additional expression study has revealed downregulation of p-PI3K, p-AKT, and p-CREB in the hippocampus of the omethoate-exposed mice[19]. These studies, thus, reveal that interaction between the PI3K/AKT pathway and CREB influences the outcome of pesticide-associated neurotoxicity [67].

### cAMP/PKA pathway

It has been known through previous studies that CREB activation can also occur through the calcium–calmodulin kinase-dependent pathway through the PLC pathway. PLC pathway activation as a result of phosphorylated Tyr816 residue, in turn, generates IP3 (Inositol triphosphate) and DAG (Diacylglycerol). Plc/Ip3 cascade leads to calcium release from internal cellular stores, initiating CaMK (Calcium/calmodulin-dependent protein kinase), while DAG activates PKC, regulating neuronal plasticity [68]. These pathways play a role in dendritic projection, branching, and expand the dendrite's thickness, neuronal survival, synaptic plasticity, cognitive activity, and differentiation [69, 70]. Pesticides like chlorpyrifos exert neurotoxic effects by dysregulating the PKA phosphorylation pathway and thereby altering the dopamine metabolite level and leading to hyperphosphorylation of tau [67].

### CREB and the RAS/MEK/ERK and RAS/MAPK pathway

However, another pathway involving RAS/RAF/MEK/ERK and RAS/MAPK signaling is also activated upon exposure to certain pesticides. RAS/MEK signaling pathway transduces signals to the cytoplasm and nucleus from its membrane receptors [71]. Major genes involved in this pathway are RAF (Rapidly accelerated fibrosarcoma kinases), MEK, MAPK, and ERK (extracellular-signal-regulated kinase). They are essential for several biological functions, including cell proliferation and differentiation. RAS/MEK pathway is crucial for promoting cognitive activity such as learning and memory formation, synaptic plasticity, and neuronal survival [72]. Impairments in spatial learning and the diminished number of neurons in the hippocampus have been attributed to decreased phosphorylated ERK 1/2 and CREB proteins.

It has been observed that rotenone-induced dopaminergic apoptosis occurs through the activation RAS/MEK pathway [73]**.** RAS gene is essential for its role in long-term potentiation (LTP) and development and the formation of memories in the central nervous system. When there is abnormal RAS signaling, it leads to the deterioration of hippocampus LTP, resulting in chronic neurotoxicity [74]. A study found that pesticide residue avermectin induces neurotoxicity by activating the RAS /RAF/MEK/ERK pathway [75]. Atrazine caused a significant downregulation in the mRNA and the protein expression levels of the MEK/ERK/CREB pathway in the rat hippocampus [50].

MEK, a mitogen-activated protein kinase, plays a primary role in the molecular process of brain progression, neuronal plasticity, long-term memory, hippocampal development, and cellular survival [76]. Studies have shown that the MEK gene can be stimulated by toxicants, including organophosphorus and organochlorine pesticides regulating apoptotic signaling cascades [77]. Chlorpyrifos insecticide-induced MEK activation resulted in ROS production and led to neuronal apoptosis [78]. Insecticides belonging to synthetic pyrethroids have a detrimental effect on cellular growth, mediated through  the MEK pathway. The activation of MEK is also involved in the long-term hippocampus potentiation, which is accountable for learning and memory formation [79]. The central role of MEK in promoting cellular stress mechanisms can be considered therapeutic target in the treatment of pesticide-induced diseases [80].

RAS protein is bound in the intrinsic part of the cellular membrane and has an internal GTPase function, which controls cell functions and stimulates the downstream kinases that belong to the mitogen-activated protein kinase pathway (MAPK) [81]. Rotenone-induced neurodegeneration develops through the upregulation of MEK that plays a role in neuron inflammation and apoptosis. In vitro experiments indicated that ROS generation induced by rotenone exposure is through the activation of p38MAPK [82]. Jun N-terminal kinase (JNK), a subfamily of MAPK and p38MAPK, was activated upon paraquat treatment, signaling the dopaminergic cell death in the SK-DAT cell line expressing sodium-dependent dopamine transporter [83, 84]. Thus, CREB activity is closely interlinked with the RAS/MEK/ERK pathway and can affect essential neuronal functions if altered from their normal levels.

### PLC/PKC signaling pathway

PLC/PKC pathway activation is crucial for synaptic remodeling, learning, and memory development. The DAG and PKC are two of the essential genes involved in this signaling [85] that result in the phosphorylation of CREB. Studies have shown that PLC/PKC pathway is activated during various toxic insults. Phospholipase C (PLC) is involved in various physiological mechanisms, such as differentiation, survival, cell proliferation, neuron maturation, and formation of appropriate neuronal circuits for the activity of the brain. Several studies prove that PLC plays a role in neurotrophin signaling cascade and numerous neuronal activities, including neurite projection, synaptic plasticity, and neuron cellular migration [86]. They are also essential in transducing signals for events such as apoptosis, autophagy, differentiation and cell cycle entrance [87]. Activation of PLC results in the release of Ca^2+^ from internal cellular stores, which later stimulates entry through the plasma membrane [88]. The irregular functioning of PLC is assumed to cause neurotoxicity by disturbing synaptic transmission and is reportedly reduced in neurodegenerative diseases. Abnormality in PLC is enough to damage the long-term potentiation in the hippocampus [86].

Bifenthrin insecticide can stimulate Ca^2+^ release from the endoplasmic reticulum by increasing the PLC activity. Reactive oxygen species and oxygen-free radicals regulate signal transduction with PKC, a serine/threonine-specific protein kinase interaction [89]. PKC activity is initiated in the brain of rats following treatment with pesticides such as organophosphorus (chlorpyrifos) and organochlorine (chlordane and DDT), which are known to produce oxidative damage [89]. The consequence of chlorpyrifos pesticide on the PKC expression affects the signaling cascade by altering PKC gene expression in the developing rat brain [90]. Dieldrin organochlorine insecticide promotes dopaminergic neuron apoptosis in rats by the upregulation of PKC expression [91]. A recent study proves that the neurotoxic effect of paraoxon organophosphate enhances the concentration of PKC phosphorylation in cerebellar cultured granule cell neurons resulting in neuronal cell damage [92]. Thus, the *Pkc* gene is another effective therapeutic target against the OP induced neurotoxicity.

## Conclusion

Although pesticides are manufactured explicitly to target various pests and insects, there are high chances that mammals too get inadvertently exposed to it, making these non-selective in their target. Exposure to pesticides has significantly increased in recent years because of the development of many agricultural sectors, irrigation facilities, and industrial and manufacturing areas. These pose a significant concern as they are speculated to lead to alterations in behavior and the physiology of an organism leading to adverse effects on an individual’s health. This review demonstrates the role of CREB and the related genes that are situated upstream in the signaling cascade and how they are involved in regulating the brain during various scenarios of exposure to pesticides such as Fipronil, Rotenone, parathion, malathion, chlorpyrifos, and deltamethrin, eventually resulting in neurotoxicity.

While CREB is well discussed as pertaining to its role in various neurodegenerative pathologies, the link between one of the increasingly concerning causative environmental factors, i.e., pesticides, and its ability to modulate the CREB pathway, is not well discussed. Further, the domino effect caused by the modulation of CREB phosphorylation or dephosphorylation on the other closely associated upstream pathways is also not well comprehended. Pesticides play a role in phosphorylation and regulation of gene expression of CREB and various different pathways involving protein kinases and neurotrophins. Some of these key elements, including Pi3K, AKT, RAS, MAPK, BDNF, and CREB, are significantly reduced, leading to a reduction in their specific brain function. A more thorough investigation will help us better understand the cumulative effects of multiple genes in these pathways. The current review gives a multi-faceted overview comparing the effects of different pesticides on various genes of the CREB and the associated pathways. It aims to provide a holistic outlook on the pesticides and their varied molecular targets within the pathways mentioned and improve our understanding of the role of pesticides in neurodegeneration. Alterations in each gene can dysregulate the whole cascade, thus, leading to altered behavioral and gene expression in the exposed individual. Further, the review will be helpful to other researchers in toxicology to select the key genes when looking to study the neurotoxic potential of different pesticides. It will facilitate the identification of valuable therapeutic targets in future studies. The review also helps identify the most potent neurotoxic pesticide, and researchers can design remedial measures against. Therefore, it is imperative to understand the possible targets of pesticide exposure which can serve as a useful biomarker in managing pesticide-induced neurotoxic symptoms.

## Data Availability

Not applicable.
